# A randomized trial to identify accurate and cost-effective fidelity measurement methods for cognitive-behavioral therapy: project FACTS study protocol

**DOI:** 10.1186/s12888-016-1034-z

**Published:** 2016-09-15

**Authors:** Rinad S. Beidas, Johanna Catherine Maclean, Jessica Fishman, Shannon Dorsey, Sonja K. Schoenwald, David S. Mandell, Judy A. Shea, Bryce D. McLeod, Michael T. French, Aaron Hogue, Danielle R. Adams, Adina Lieberman, Emily M. Becker-Haimes, Steven C. Marcus

**Affiliations:** 1 Department of Psychiatry, University of Pennsylvania Perelman School of Medicine, 3535 Market Street, 3rd floor, Philadelphia, PA 19104 USA; 2 Department of Economics, Temple University, Philadelphia, PA USA; 3 National Bureau of Economic Research, Cambridge, MA USA; 4 Institute for the Study of Labor (IZA), Bonn, Germany; 5 Department of Psychology, University of Washington, Seattle, WA USA; 6 Department of Psychiatry and Behavioral Sciences, Medical University of South Carolina, Charleston, SC USA; 7 Department of Psychology, Virginia Commonwealth University, Richmond, VA USA; 8 Department of Health Sector Management and Policy and Department of Sociology, University of Miami, Coral Gables, FL USA; 9 The National Center on Addiction and Substance Abuse, New York, NY USA; 10 School of Social Policy and Practice, University of Pennsylvania, Philadelphia, PA USA; 11 Annenberg School for Communication, University of Pennsylvania, Philadelphia, PA USA; 12 Department of Medicine, University of Pennsylvania, Philadelphia, PA USA

**Keywords:** Treatment fidelity, Evidence-based practice, Implementation, Cost-effectiveness

## Abstract

**Background:**

This randomized trial will compare three methods of assessing fidelity to cognitive-behavioral therapy (CBT) for youth to identify the most accurate and cost-effective method. The three methods include self-report (i.e., therapist completes a self-report measure on the CBT interventions used in session while circumventing some of the typical barriers to self-report), chart-stimulated recall (i.e., therapist reports on the CBT interventions used in session via an interview with a trained rater, and with the chart to assist him/her) and behavioral rehearsal (i.e., therapist demonstrates the CBT interventions used in session via a role-play with a trained rater). Direct observation will be used as the gold-standard comparison for each of the three methods.

**Methods/design:**

This trial will recruit 135 therapists in approximately 12 community agencies in the City of Philadelphia. Therapists will be randomized to one of the three conditions. Each therapist will provide data from three unique sessions, for a total of 405 sessions. All sessions will be audio-recorded and coded using the Therapy Process Observational Coding System for Child Psychotherapy-Revised Strategies scale. This will enable comparison of each measurement approach to direct observation of therapist session behavior to determine which most accurately assesses fidelity. Cost data associated with each method will be gathered. To gather stakeholder perspectives of each measurement method, we will use purposive sampling to recruit 12 therapists from each condition (total of 36 therapists) and 12 supervisors to participate in semi-structured qualitative interviews.

**Discussion:**

Results will provide needed information on how to accurately and cost-effectively measure therapist fidelity to CBT for youth, as well as important information about stakeholder perspectives with regard to each measurement method. Findings will inform fidelity measurement practices in future implementation studies as well as in clinical practice.

**Trial registration:**

NCT02820623, June 3rd, 2016.

## Background

Research to improve client outcomes in community mental health has been hindered by an inability to accurately and inexpensively measure fidelity [1]. Fidelity includes adherence, or how closely the components of a protocol are followed, and competence, or how skillfully the components are implemented and how responsive the therapist is to the client and situation [2]. Fidelity has been identified in implementation science frameworks as the mechanism by which desired outcomes are achieved [3–5], and as an indicator of quality of care [6]. Stakeholders in behavioral health including researchers [7], funders [8], policy makers, and agency leaders (e.g., supervisors, clinical directors) agree upon the importance of measuring fidelity. However, fidelity measurement in the community is challenging because there are few instruments that are both efficient and have demonstrated reliability and validity [9].

Direct observation of therapist behavior, considered by some to be the gold standard for measuring fidelity to psychosocial treatments, requires extensive resources. When fidelity is measured in community settings, which occurs rarely, the most commonly used method is therapists’ self-report [10]. Unfortunately, concordance between observation and self-report is low [11]. There is a critical need to identify and evaluate methods of fidelity measurement that are both accurate (i.e., measure what they intend) and cost-effective [7]. Innovative alternatives from other disciplines, particularly medicine, may present advantages for the measurement of fidelity accurately and cost-effectively [10, 12–16].

### Objectives and aims

Our long-term research goal is to strengthen the public health impact of psychosocial treatments by identifying methods to accurately measure fidelity that are well suited for use in both research and practice. The objective of this project, “Fidelity Accuracy: Comparing Three Strategies” (Project FACTS) is to compare the accuracy, costs, and cost-effectiveness of three methods to measure fidelity to cognitive-behavioral therapy (CBT) for youth, an established evidence-based practice [17]. Three fidelity measurement conditions (self-report; chart-stimulated recall; and behavioral rehearsal) will be compared to direct observation. Self-report refers to therapist completion of a brief questionnaire with regard to CBT interventions used with a client in session [9]. To address the limitations of self-report, we will ensure that each CBT intervention is clearly defined and therapists will be trained in how to rate their own fidelity. Chart-stimulated recall refers to a brief structured interview with a therapist about a client session during which the therapist reviews the client’s file to aid recall of specific CBT interventions used [18]. Behavioral rehearsal, also known as standardized patient methodology, refers to a role-play between a therapist and a trained actor where the therapist demonstrates their use of CBT interventions [10]. We are most interested in the potential of the two innovative methods used in the medical literature (i.e., chart-stimulated recall and behavioral rehearsal) that may represent a reasonable compromise between direct observation and self-report and provide resolution to the fidelity measurement quandary of the past two decades.

We will randomize 135 therapists delivering CBT in community mental health agencies to each of the three fidelity measurement method conditions. We will compare the method in each arm to direct observation of fidelity using the Therapy Process Observational Coding System for Child Psychotherapy-Revised Strategies scale (TPOCS-RS) [19] as the gold-standard comparison. Specifically, we propose to:

1. Identify the most accurate fidelity measurement method. We hypothesize chart-stimulated recall and behavioral rehearsal will be more accurate than self-report when each is compared to direct observation.
2. Estimate the economic costs and cost-effectiveness of the proposed fidelity measurement techniques. We hypothesize chart-stimulated recall and behavioral rehearsal will be more cost-effective than self-report, given the hypothesized greater accuracy of chart-stimulated recall and behavioral rehearsal.
3. Compare stakeholder motivation to use each method, and identify their perceived barriers and facilitators to using each method. We hypothesize stakeholders will be most motivated to use chart-stimulated recall in future endeavors, because it is most akin to existing supervision practices.

## Methods/design

### Aim 1: identify the most accurate fidelity measurement method

Project FACTS evaluates the accuracy of three fidelity measurement methods in comparison to direct observation: self-report, chart-stimulated recall, and behavioral rehearsal. Self-report may be the least accurate method for several reasons. First, therapists may not understand the meaning of questions that appear on such measures. For example, one item from a commonly used self-report instrument, “Did you use systematic desensitization, with imagined or real exposure to feared objects or situations, with your client?” [20] includes several technical terms. Therapists may use these interventions but not know what they are called, leading to under-reporting. Second, therapists may over-endorse use of CBT interventions due to social desirability demands. Third, therapists treat many clients each week, and their recall of session content may be poor if they do not complete forms immediately following the session.

Chart-stimulated recall was developed as a clinical assessment tool in medicine, where it has demonstrated reliability and validity with respect to physician behavior [12–16]. Chart-stimulated recall is an interviewing technique used to measure the process and quality of clinical decision making in physicians. During this interview, typically conducted with a supervisor, physicians use the patient chart to stimulate their memory of their treatment plan [16]. To date, chart-stimulated recall has not been used in mental health. We hypothesize chart-stimulated recall will allow for accurate fidelity measurement because it reduces recall bias in two ways. First, access to the chart may allow therapists to remember details they would have otherwise forgotten. Second, the presence of a trained individual^1^ facilitates clarity of recall via the use of standardized probes (e.g., defining key terms). Finally, we hypothesize that chart-stimulated recall will be superior to other methods in capturing adherence because of its emphasis on specific treatment interventions and the capacity of the interviewer, trained to identify such interventions, to accurately determine whether or not particular CBT intervention was used.

Behavioral rehearsal, also called standardized patient methodology, is a well-established method in medicine for evaluating physician clinical practice [21], and has demonstrated promise in assessing psychosocial intervention fidelity in preliminary work [10]. Behavioral rehearsal refers to a role-play between a therapist and trained individual in which a therapist demonstrates the CBT interventions used in session with their client. We hypothesize this method is effective because it allows therapists to show what they did in the treatment session (e.g., conducting a specific cognitive-behavioral intervention, such as exposure). Further, we hypothesize behavioral rehearsal will be superior in capturing competence because the trained individual observing the therapist role-playing particular CBT interventions evaluates the therapist’s skillfullness and responsiveness, which represent core competence domains [22].

The objective of Aim 1 is to identify the most accurate fidelity measurement method. Specifically, we define accuracy as criterion validity (i.e., how well each measurement method captures the dimensions of fidelity—adherence and competence—compared with direct observation). We hypothesize both chart-stimulated recall and behavioral rehearsal will be more accurate than self-report in capturing overall fidelity (i.e., adherence and competence). Further, we hypothesize chart-stimulated recall will best capture adherence, whereas behavioral rehearsal will best capture competence.

#### Study design

We use a parallel group randomized trial design with equal allocation for this measurement study.

#### Study setting

The City of Philadelphia’s Department of Behavioral Health and Intellectual disAbility Services (DBHIDS) is committed to integrating evidence-based practice in the public mental health system. We will conduct our study within the context of ongoing initiatives to support the adoption and implementation of evidence-based practice (EBP) in the City of Philadelphia, supported by DBHIDS. Since 2007, DBHIDS has trained more than 300 therapists in CBT, an established EBP for the treatment of a number of child and adult psychiatric disorders. DBHIDS provides a structured therapist and supervisor training program for initiatives in cognitive therapy (Beck Community initiative), trauma-focused CBT (Philadelphia Alliance for Child Trauma Services Initiative), prolonged exposure, and dialectical behavior therapy. The training and ongoing consultation provided to therapists and supervisors closely follows treatment developers’ recommendations and includes consultation. For example, therapists and supervisors in the Beck Community Initiative participate in 22 h of workshops followed by 6 months of weekly consultation with the treatment development team [23].

#### Human subjects protection

All procedures have been approved by the City of Philadelphia and University of Pennsylvania IRB.

#### Participants

Participants include community mental health agencies, therapists, their youth clients (7 to 24 years), and their guardians. We include young adults up to age 24 because agency leadership indicated that this is the age range served in their settings under the umbrella of child and family services.

#### Community mental health agencies

We will recruit community mental health agencies that are participating in at least one of two initiatives to implement CBT for youth (i.e., Beck Community Initiative [23] and the Philadelphia Alliance for Child Trauma Services Initiative [24]). Approximately 30 community mental health agencies that serve youth through their outpatient programs have participated in at least one of the initiatives. To recruit agencies, we will contact agency leadership to ascertain interest in participating, as we have done in previous studies. We will enroll approximately 12 organizations until we reach the target number of therapists [25].

#### Therapists

**Fig. 1:**
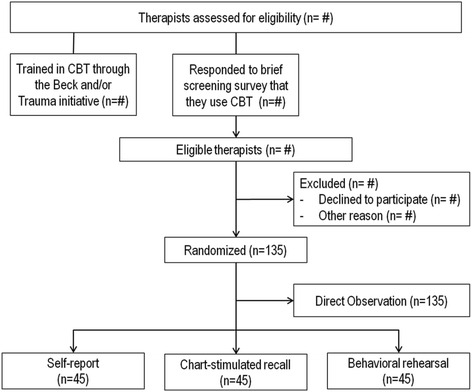
CONSORT diagram

We will recruit therapists employed at the community mental health agencies participating in CBT implementation initiatives. Because we are interested in how well each measurement method captures fidelity to CBT, to be eligible, therapists must implement CBT interventions with some of their clients. Therapists at participating agencies will be eligible for study participation: (a) if they are formally participating in the CBT initiatives as trainees, OR (b) if they report delivering CBT to clients (See Fig. 1 for CONSORT diagram). With regard to therapists formally participating in a CBT initiative, there is an expectation as part of their participation in the initiative that they will deliver CBT to clients on their caseload. With regard to therapists not enrolled in CBT initiatives who report delivering CBT, we will ask them to rate their use of CBT interventions (based upon the TPOCS-RS [19]) on a 7-point Likert scale. We will also ask them if they believe they will be able to identify at least three clients in a month that they treat using CBT. Those who endorse using CBT and an ability to identify at least three clients on their caseload with whom they use CBT will be eligible for study participation. We anticipate that therapists will be enrolled in the study for approximately 3 weeks.

#### Youth participants

Therapists in Philadelphia typically have approximately 30 client sessions per week [26]. We plan to obtain three unique sessions (i.e., one session from three separate clients) per therapist for a total of 405 youth client sessions. CBT interventions differ during the course of treatment (for example, treatment of anxious youth typically begins with sessions focused on psychoeducation and rapport building and progress to exposure later in treatment [27]). Accordingly, session number is the basis of the sampling plan. We will randomly select sessions that occur early (sessions 2–9) and late (sessions 10 and higher) in treatment.^2^ Any randomly selected youth (7 to 24 years) who is in treatment for any psychosocial difficulties may participate. Youth in foster care, and youth who do not speak English and/or have a primary caregiver who does not speak English will not be eligible to participate in the study. Youth/parent participation is limited to allowing the research team to audio-record one treatment session. Youth will be paid $10 for allowing their treatment session to be audio-recorded. Youth participation will be complete after their treatment session is recorded.

#### Randomization

Eligible therapists will be recruited at a study launch meeting at each agency. At that meeting, the manager of research projects, with the assistance of the biostatistician (SCM), will randomize therapists into one of the three measurement arms ensuring that proper allocation concealment guidelines are followed. To maintain balance, we will ensure that we have equal numbers of therapists in each study arm in each site (i.e., equal randomization). We will use stratified sampling by years of training experience to ensure we account for variability in therapist experience that may impact performance. The randomization process will not be blinded.

#### Procedure

The research team will work with therapists and staff at each agency to identify potential client sessions. Therapists or agency staff will provide the research team with a de-identified schedule each week for each participating therapist. Three clients per therapist will be randomly selected using stratified sampling by session number. Therapists will be asked by the research team to approach each randomly selected client and caregiver about potential participation in the project. After a youth and caregiver provide verbal permission, research assistants, who will be on-site, will meet with them to ascertain their interest in study participation and obtain assent and consent. The session will be recorded using a digital audio-recorder and will be uploaded to an encrypted hard drive immediately following the session by the research team. Therapists randomized to the self-report condition will complete the self-report measure within 48 h of their session. Therapists randomized to the chart-stimulated recall condition will complete their interview within 1 week. Therapists randomized to the behavioral rehearsal condition will complete their role-play within 1 week.

#### Direct observation

All therapists will have the three study therapy sessions recorded using a digital audio-recorder. Subsequently, all three sessions will be coded using the TPOCS-RS [19]. The scores to emerge from the TPOCS-RS will serve as the gold-standard fidelity measurement comparison. We will use the TPOCS-RS to rate adherence and competence^3^ for cognitive and behavioral interventions (e.g., cognitive education, operant strategies) in each of the sessions. The TPOCS-RS cognitive and behavioral items have demonstrated strong score reliability and validity [19]. We will train research assistants to adequate inter-rater reliability at the item level (ICC > .60) prior to rating sessions. We will continually monitor reliability over the course of the project to avoid rater drift.

#### Self-report

Therapists will complete the TPOCS-CBT Self Report scale (TPOCS CBT-SR) for each of their three recorded sessions, using REDCap, a HIPAA-compliant electronic data capture system. The TPOCS CBT-SR will index the occurrence of the CBT interventions assessed by the TPOCS-RS (see above) and will be created in collaboration with the TPOCS-RS developer (BDM). Therapists will rate both their adherence and competence to the CBT interventions in each session. To circumvent two major challenges of self-report, difficulty understanding items and lack of training in how to judge behavior, we will: (a) provide an operational definition for each item on the TPOCS CBT-SR (e.g., behavioral activation: teaches the relationship between pleasant or activating activities and improvement in mood, employs pleasant or activating experiences in session to demonstrate the impact on mood, or assigns participation in a pleasant or activating event with the expressed purpose of improving mood), and (b) provide therapists with a 30-min training session that includes sample vignettes of particular behaviors and information about how those vignettes should be rated.

#### Chart-stimulated recall

Therapists will be asked to bring the charts of the three youth whose sessions were recorded to the chart-stimulated recall interview. The interview, conducted by a research assistant trained in both chart-stimulated recall methodology and CBT, is structured as follows for each session. The interviewer will prompt therapist recall with an open-ended question (*“Talk me through your last session with your client. Tell me what you did.”*). While the therapist is speaking, the interviewer documents any elements that represent a prescribed CBT intervention on a worksheet. The interviewer privately reviews a list of the CBT interventions based upon the TPOCS-RS and verbalizes probes to determine whether the therapist completed any of the interventions. When necessary, the interviewer asks follow-up questions to explore the degree to which, and how skillfully, the interventions were used. We will train research assistants to adequate inter-rater reliability prior to conducting chart-stimulated recall interviews. Research assistants will code these interactions as they occur; all interviews will be recorded and 20 % will be coded by an independent evaluator for purposes of inter-rater reliability.

#### Behavioral rehearsal

Therapists will attend a 1-h meeting with a trained research assistant to conduct behavioral rehearsal for the three recorded sessions. The research assistant will provide the therapist with a list of the TPOCS-RS CBT interventions and ask him/her to identify all of the CBT interventions used in each of their recorded sessions. The research assistant will then ask the therapist, “*Please role-play how you used each CBT intervention in session with your client.”* The therapist will then role-play each CBT intervention used. We will train research assistants to adequate inter-rater reliability prior to conducting behavioral rehearsals with therapists. Research assistants will code these interactions as they occur; all interviews will be recorded and 20 % will be coded by an independent evaluator for inter-rater reliability.

#### Power analysis

Our power calculations balance scientific demands with the logistical and financial realities of community-based research. We will conduct the study in approximately 12 agencies; and, within these agencies, randomize therapists to one of three fidelity measurement conditions. Statistical power calculations were conducted to identify the number of therapists needed to test the null hypothesis of no difference between direct observation and the assigned fidelity measurement method across study arms. To quantify the magnitude of a detectable effect, we use Cohen’s *d*, a measure of the difference in mean outcomes between two groups in standard deviation units. This unit-less measure provides a convenient metric for examining relative effect sizes in our study. Cohen’s *d* of 0.2, 0.5, and 0.8 are conventionally considered to be “small,” “medium,” and “large” effects, respectively [28]. We used the CRT-Power software package to calculate sample sizes needed to achieve 80 % power (alpha = 0.05) to detect these effects in our 2-level hierarchical design where therapists are nested within agencies and clients are nested within therapist. The software accounts for this nesting using the Intracluster Correlation Coefficient (ICC), a measure of the relatedness of clustered data that compares the variance within and between clusters. The ICC in our previous work was 0.23 [26]. With 45 therapists in each of the three conditions, we will have sufficient power to detect a Cohen’s *d* of .56 if such a difference exists.

#### Data analysis

All analyses are intent-to-treat. Differences across the three arms of the study will be assessed with mixed-effects multivariate regression models where the dependent variable for each session is the difference in score between direct observation and the specific fidelity method under consideration. All four fidelity measurement methods include CBT interventions which are scaled identically, thus facilitating comparison. We will measure the dependent variables in three ways: raw score adherence, raw score competence, and total fidelity *t*-score (composite of adherence and competence raw scores). Our regression models will include fixed effects for fidelity measurement method and site along with a random effect for therapist to account for the nesting of client session within therapists. Although we expect our study sites to be demographically and clinically homogeneous, we include site as a fixed effect in the model to account for any unexpected variation. Our primary interest is in the beta coefficient for the fidelity measurement method, which tells us whether there is any difference in accuracy relative to direct observation between the intervention arms. Our first hypothesis is that the total fidelity *t*-score will be more accurate for both chart-stimulated recall and behavioral rehearsal than self-report. Our second hypothesis is that chart-stimulated recall will capture adherence best (i.e., most accurate relative to TPOCS-RS adherence score). Our third hypothesis is that behavioral rehearsal will capture competence best (i.e., most accurate relative to TPOCS-RS competence score). To test these hypotheses, we will use the model described above to examine whether the mean of the dependent variable differs for each pair-wise comparison of our intervention arms. We will then repeat that analysis after transforming the dependent variable into *t*-scores to allow computation of standardized differences for each pairwise comparison, so that we can rank accuracy [28]. A second set of regression models will be conducted that includes an additional fixed-effect term for time and an interaction (time X fidelity measurement method), allowing us to examine if there are changes in outcome over time by measurement method. Evidence of such changes could reflect differential practice effects associated with the fidelity measurement conditions.

### Aim 2: estimate the economic costs and cost-effectiveness of the proposed fidelity measurement techniques

The primary objective of this aim is to use economic evaluation methods to estimate the costs and cost-effectiveness of the fidelity measurement methods. Our hypothesis is that chart-stimulated recall and behavioral rehearsal will have higher total and average economic costs compared to self-report, but that these conditions will be more cost-effective given their hypothesized greater accuracy relative to self-report. We will also generate economic costs of direct observation but we will not calculate cost-effectiveness because it serves as our baseline measure of accuracy. Although use of these economic evaluation methods is widespread in medicine [29], they are rarely utilized in implementation studies [5]. To this end, the protocol developed in this application can serve as a template for future implementation studies that seek to examine economic costs and cost-effectiveness.

#### Effectiveness (i.e., accuracy) estimation

Our measure of accuracy in this study is the difference in accuracy score (i.e., total fidelity *t*-score, adherence raw score, competence raw score) between direct observation and the specific fidelity measurement method.

#### Cost estimation

Economic costs will be collected in two ways. The first approach uses a modified version of the Drug Abuse Treatment Cost Analysis Program (DATCAP), a comprehensive cost instrument used to collect detailed information on resources used in behavioral health settings. The data analysis algorithms for the DATCAP are well-established and have been tested extensively in numerous studies, including mental health services interventions [30–39]. DATCAP resource categories that are relevant for the present study include: the value of therapist time to complete the fidelity measurement methods (i.e., their hourly wage), the physical space allocated to completing the fidelity measurement method, financial incentives paid to participants, and other resources (e.g., computers, office supplies,) used to develop and complete the fidelity measurement method. Economic or opportunity costs will reflect the fair market value of all resources.

The second approach to cost estimation will adopt a community mental health agency perspective. Namely, we are interested in capturing the direct costs incurred by a community mental health agency in the course of implementing each fidelity measurement method as these are the costs that such an agency would likely consider when deciding whether or not to implement such a measurement. We expect these costs to pertain mostly to therapist time and other miscellaneous resources (e.g., computers, office supplies). Our intent is to estimate the economic costs that would be incurred by a community mental health agency if they were to incorporate one of the fidelity measurements evaluated in this study. Thus, purely research costs will be excluded (e.g., financial incentives paid to therapists or families), which is standard practice in economic evaluations when replicating methods in community-based settings.

#### Procedure

The research team will provide the modified DATCAP [40] to agency leadership most familiar with the operations and financing of the program. After these personnel have reviewed the materials, conference calls will be conducted between these leaders and the researchers to formulate strategies for preliminary data collection and to answer questions. The researchers will provide agency leadership with guidance regarding the type and source of resource use and cost information to gather. Data will be gathered using REDCap.

#### Cost effectiveness analysis

We will estimate and compare ratios of cost and effectiveness (accuracy as estimated in Aim 1) for sessions across study conditions. The ratios will be compared using the following formula for each fidelity method:

$$ Average\  Cost\  Effectiveness\  Ratio=\left( Average\  cost\right)/\left( Average\  score\  difference\right) $$

where *Average cost* is the average cost per session when using a specific fidelity measurement method, and *Average score difference* is the average difference in score per session between direct observation and the specific fidelity measurement method. This ratio represents the average cost per session of achieving a one-unit improvement in score between direct observation and the specific fidelity measurement method under consideration (i.e., improved accuracy). The fidelity measurement method that produces the lowest ratio will be deemed the most cost-effective. We will conduct these analyses for each of the three accuracy scores produced in Aim 1 (adherence raw score, competence raw score, total fidelity *t*-score).

### Aim 3: compare stakeholders’ motivation to use each method, and identify their perceived barriers and facilitators to use of each

In addition to the potential contribution that the identification of accurate and cost-effective fidelity measurement methods can make to the broader implementation science literature, this work will also produce pragmatic measures [41] that can be useful for community mental health agencies. These fidelity measurement methods can be integrated as quality assurance tools by supervisors during supervision, given the literature suggesting that most therapists receive supervision in community mental health [26, 42, 43]. Investigating factors that may affect eventual implementation of the fidelity measurement methods of interest in community mental health represents an important next step. This aim provides a truly community-based approach by studying the perspectives of the stakeholders themselves [44].

Research suggests that a number of multi-level factors affect successful implementation [3–5]. In the current study, we are particularly interested in understanding individual motivation as a potential facilitator of implementation of the three fidelity measurement methods given a large literature in social psychology demonstrating that one’s motivation to perform a particular behavior predicts whether that behavior is performed [45]. If study findings show a particular method is accurate but therapists or supervisors have low levels of motivation to use it, it will be important to understand why motivation is low in order to understand how to increase it. Extensive evidence shows that motivation can be strengthened by theory-based efforts designed to target perceived barriers and facilitators [46, 47]. Therefore, the primary objectives of this exploratory aim are to (a) measure therapist and supervisor motivation to use each fidelity measurement method and (b) identify perceived barriers and facilitators that influence motivation for each method.

#### Procedure

We will use a mixed-methods approach. Using survey methods and tools developed and validated in the social psychology literature [48], we will quantitatively measure and compare therapist and supervisor level of motivation for each method. These measures have demonstrated predictive, face, and construct validity [45]. Subsequently, among a subset of participants, we will conduct semi-structured interviews to assess perceived barriers and facilitators to using each method.

#### Participants

Participating therapists will be asked to complete a brief quantitative survey of motivation as part of their participation in Aim 1. Additionally, we will use purposive sampling to recruit 48 participants (i.e., 36 therapists [12 therapists from each of the three conditions] and 12 supervisors) for semi-structured interviews [49]. We selected 12 per group because prior research suggests it to be an appropriate number for thematic saturation [49].

#### Quantitative instrument

Motivation will be quantitatively measured for each fidelity measurement method using a 7-point response option scale. We will ask therapists about their motivation to use the various fidelity measurement methods (e.g., “*Imagine that your supervisor uses behavioral rehearsal about once a month with you to evaluate your fidelity to CBT. How likely are you to participate in this type of supervision*?”) The response options range from “*Very unlikely*” to “*Very likely*.” Each therapist will also be asked to report their motivation towards using the fidelity method that s/he has been randomly assigned. Supervisors will be asked about their motivation to use the fidelity methods (e.g., “*If you and your agency agreed to use chart-stimulated recall to evaluate therapist fidelity to CBT, how likely are you to use it at least once a month*?”).

#### Qualitative semi-structured interviews

Semi-structured interviews will be conducted with a subset of participants. The interviews will include validated, standard open-ended questions to elicit perceived barriers and facilitators to the use of a particular fidelity measurement method. For example, we will ask stakeholders to share their perceived advantages and disadvantages to using a particular method (e.g., “*Let's start with the bad things that can happen if you were to regularly use [method]. What are some of them*?”). We will also ask who they believe will approve and disapprove of their use of a particular method, and what would make it difficult or easy to use a particular method. All of the measures will be piloted and cognitive response testing will be used to identify ambiguous wording [50].

#### Quantitative analysis

We will repeat the analyses described in Aim 1 (see Data Analysis section of Aim 1) using the motivation score as the dependent variable. These mixed effects regression models will evaluate the extent to which motivation to implement differs by fidelity measurement method.

#### Qualitative analysis

The analyses, conducted separately for therapists and those in leadership positions, will identify the most commonly reported perceived barriers and facilitators to the use of each fidelity measurement method. We will follow standardized procedures to create mutually exclusive and exhaustive coding categories for beliefs [51]. Trained staff will independently review and code transcripts to identify and abstract all reported barriers and facilitators following standardized procedures. We will ensure interrater agreement reaches and is maintained at 80 %. For any inconsistencies, the principal investigator will make a final determination. For each question asked of each fidelity measurement method, we will determine the proportion of respondents that mentioned each barrier and facilitator.

## Discussion

### Innovation

Few fidelity measurement methods that are both accurate and cost-effective have been identified. The current study is innovative because it proposes to identify accurate and cost-effective methods to measure fidelity. We will investigate two innovative methods that may represent a reasonable compromise between direct observation and self-report (i.e., chart stimulated-recall and behavioral rehearsal). Other innovations include: (1) a strong partnership between our research team and community stakeholders, whose needs drive the research question [44]; (2) our goal of identifying a method that stakeholders can use in future implementation efforts; (3) our understanding of factors influencing stakeholder motivation to use fidelity measurement methods; (4) the inclusion of a cost-effectiveness measure, an understudied implementation outcome [5]; (5) and the generation of generalizable information on best practices for fidelity measurement in CBT, the most widely used evidence-based practice.

### Limitations

Despite the methodological strengths of this fidelity measurement study, there are limitations. First, we will not measure client outcomes and thus will not be able to assess relations among the various measurement methods and client outcomes. Second, fidelity is a multifaceted construct and we are only measuring two of the facets (adherence and competence). We will not attempt to index sequencing of CBT interventions or the targets of treatment in our assessment of fidelity. In other words, when rating fidelity, we will not take into account whether or not the “right” CBT intervention was used for the child’s presenting diagnosis (e.g., exposure for anxiety disorders) or if the therapist intended to do one CBT intervention but ended up using another one. These are more sophisticated questions that warrant further investigation.

### Impact

The study of fidelity measurement methods has important implications for the progress of the implementation science field, treatment quality more broadly, and ultimately the public health impact of evidence-based psychosocial treatments. At the completion of this study, we hope to have identified accurate and cost-effective fidelity measurement methods that can be used in research and practice. Additionally, we will explore stakeholder motivation to use these methods. This information gathered will be used in future implementation trials and be used practically by supervisors in community settings for quality assurance, thus serving multiple purposes.

## Abbreviations

CBH: Community Behavioral Health
CBT: Cognitive-behavioral therapy
DATCAP: Drug Abuse Treatment Cost Analysis Program
DBHIDS: Department of Behavioral Health and Intellectual disAbility Services
EBP: Evidence-based practice
FACTS: Fidelity Accuracy: Comparing Three Strategies
HIPAA: Health Insurance Portability & Accountability Act
ICC: Intracluster Correlation Coefficient
NIMH: National Institute of Mental Health
REDCap: Research Electronic Data Capture
TPOCS-CBT-SR: Therapy Process Observational Coding System for Child Psychotherapy-CBT Self Report scale
TPOCS-RS: Therapy Process Observational Coding System for Child Psychotherapy-Revised Strategies scale

## Footnotes

1 For the purposes of this study, the trained individual refers to research assistants who will conduct chart-stimulated recall and behavioral rehearsal.

2 We elected not to include session 1 because it is usually oriented toward assessment and rapport building, rather than active intervention.

3 Note, the TPOCS-RS does not currently contain a competence rating. We will develop this as part of this project.
